# Viral Infection and Respiratory Exacerbation in Children: Results from a Local German Pediatric Exacerbation Cohort

**DOI:** 10.3390/v14030491

**Published:** 2022-02-27

**Authors:** Erwan Sallard, Frank Schult, Carolin Baehren, Eleni Buedding, Olivier Mboma, Parviz Ahmad-Nejad, Beniam Ghebremedhin, Anja Ehrhardt, Stefan Wirth, Malik Aydin

**Affiliations:** 1 Center for Biomedical Education and Research (ZBAF), Department of Human Medicine, Faculty of Health, Institute of Virology and Microbiology, Witten/Herdecke University, 58453 Witten, Germany; erwan.sallard@uni-wh.de (E.S.); anja.ehrhardt@uni-wh.de (A.E.); 2 Center for Child and Adolescent Medicine, Center for Clinical and Translational Research (CCTR), Helios University Hospital Wuppertal, Witten/Herdecke University, 42283 Wuppertal, Germany; frank.schult@helios-gesundheit.de (F.S.); olivier.mboma@uni-wh.de (O.M.); stefan.wirth@uni-wh.de (S.W.); 3 Laboratory of Experimental Pediatric Pneumology and Allergology, Center for Biomedical Education and Research, Faculty of Health, School of Life Sciences (ZBAF), Witten/Herdecke University, 58455 Witten, Germany; carolin.baehren@uni-wh.de (C.B.); eleni.buedding@uni-wh.de (E.B.); 4 Institute for Medical Laboratory Diagnostics, Center for Clinical and Translational Research (CCTR), Helios University Hospital Wuppertal, Witten/Herdecke University, 42283 Wuppertal, Germany; parviz.ahmad-nejad@helios-gesundheit.de

**Keywords:** human rhinovirus, respiratory syncytial virus, virus, infection, asthma, bronchitis, exacerbation, children

## Abstract

Respiratory viruses play an important role in asthma exacerbation, and early exposure can be involved in recurrent bronchitis and the development of asthma. The exact mechanism is not fully clarified, and pathogen-to-host interaction studies are warranted to identify biomarkers of exacerbation in the early phase. Only a limited number of international exacerbation cohorts were studied. Here, we have established a local pediatric exacerbation study in Germany consisting of children with asthma or chronic, recurrent bronchitis and analyzed the viriome within the nasopharyngeal swab specimens derived from the entire cohort (*n* = 141). Interestingly, 41% of exacerbated children had a positive test result for human rhinovirus (HRV)/human enterovirus (HEV), and 14% were positive for respiratory syncytial virus (RSV). HRV was particularly prevalent in asthmatics (56%), wheezers (50%), and atopic (66%) patients. Lymphocytes were decreased in asthmatics and in HRV-infected subjects, and patients allergic to house dust mites were more susceptible to HRV infection. Our study thus confirms HRV infection as a strong ‘biomarker’ of exacerbated asthma. Further longitudinal studies will show the clinical progress of those children with a history of an RSV or HRV infection. Vaccination strategies and novel treatment guidelines against HRV are urgently needed to protect those high-risk children from a serious course of disease.

## 1. Introduction

Asthma is still one of the most common respiratory diseases [1,2], with 262 to 340 million cases worldwide, including 800,000 affected children living in Germany and a region-dependent incidence [3,4,5,6,7,8] associated with a high number of undiagnosed cases [9,10,11,12]. Based on the German national guidelines, the NVL Asthma 2020 (=Nationale Versorgungsleitlinie Asthma 2020), severe asthma exacerbation is defined by tachypnea, tachycardia, peak expiratory flow variations, reduced oxygen saturation, or speech dyspnea [13]. In addition to environmental factors [14,15], viral infections also play a leading role in asthma exacerbation [16,17,18,19,20]. Depending on the virus, an infection can influence the clinical course and may lead to an alarming increase in morbidity and mortality when appropriate interventions are not successfully initiated [21,22,23,24,25,26,27,28]. Despite vastly improved therapeutic possibilities, asthma mortality still remains tragically high with 461,000 deaths in 2019 [6].

While mortality, severity, and prevalence vary widely geographically, the prevalence of this disease in Germany is estimated to be approximately 5% in adults and 10% in children [29,30,31,32,33]. Asthma remains one of the costliest disorders and imposes a significant burden on society and patients, resulting in 11.0 million physician visits and 1.7 million emergency room visits, and is the leading cause of missed school days [29,34,35,36].

Past studies reported that human rhinovirus (HRV) [37,38,39,40] or respiratory syncytial virus (RSV) [41,42] has a significant impact on asthma. In addition, influenza virus, boca-, adeno-, or metapneumoviruses can also cause serious clinical courses in predisposed patients [43,44,45]. Moreover, HRV and RSV have been proposed to induce secondary diseases, e.g., atopic disorders whose underlying mechanism is not fully understood yet [46,47,48,49,50,51].

Exacerbation cohorts may provide useful information on pathomolecular interactions, which can be used for the definition of biomarkers [52,53,54]. However, data from exacerbation cohorts of children with asthma are still scarce [52,53,54].

Thus, a pediatric exacerbation network, including subjects with chronic recurrent bronchitis or asthma suffering from a respiratory exacerbation/deterioration of the general status were recruited, and nasopharyngeal swab specimens were obtained [52]. For this project, data of the study cohort were stratified based on demographic, clinical, and blood variables [52].

With this project, we aimed to understand the respiratory viriome in connection to microbiome results of exacerbated children. Observations from such a cohort may provide important insights into the acute phase of an asthmatic child for a better understanding of causative molecular aspects.

## 2. Materials and Methods

### 2.1. Subject Description and Sample Collection

Between the years 2017 and 2019, children and adolescents with a history of frequent bronchitis or asthma presenting symptoms of an acute exacerbation were included. Two diseased cohorts were categorized into ‘wheezers’ and ‘asthmatics’ [52]. The last subject was enrolled in 2019 before the SARS-CoV-2/COVID-19 pandemic. Therefore, a comparison between virus panel results before and during the pandemic cannot be performed. For the present study, the cohorts were redefined based on distinct clinical and molecular aspects, which will be explained below. In detail, children who were between three months and five years of age with recurrent bronchitis symptoms (‘wheezers’) and with acute respiratory complaints on the day of presentation at the study center were recruited after obtaining informed consent. According to current asthma guidelines, wheezers cannot be appropriately diagnosed as asthmatics since a lung function test (e.g., spirometry) cannot be performed to confirm the diagnosis appropriately.

Asthmatics who were five to seventeen years old and suffering from an acute exacerbation were also recruited based on the national/international recommendations for the diagnosis of asthma. Acute airway symptoms, including cough, tachy-dyspnea, chest tightness, etc., are included in the Asthma Control Test/GINA (=Global Initiative for Asthma) score (e.g., https://www.asthma.com/understanding-asthma/severe-asthma/asthma-control-test/, accessed on 10 December 2021) [52,55,56] and reflect an ‘exacerbation’ for both diseased cohorts (asthma/wheezers) in this prospective observational trial. Moreover, healthy controls between 3 months to 17 years of age without any acute febrile infection within the last few weeks before study inclusion and without any chronic disorders were also recruited [52].

The stratification of the atopic status was defined according to the following criteria: ImmunoCAP class ≥ 3, eosinophils, allergic rhino-conjunctivitis, and allergic dermatitis.

The steroid status was set as positive when a steroid or leukotriene receptor antagonist intake were present within the last months. Moreover, epidemiological and clinical parameters were recorded in order to perform correlation analyses. Among the biomaterials, nasopharyngeal swabs using Copan eSwabs^TM^ versatile liquid Amies media were deep frozen at −80 °C upon collection for further analyses [52,57].

### 2.2. FilmArray Respiratory Panel Testing

For the automated simultaneous detection of different pathogens of the upper respiratory tract (Table 1), the BioFire^®^ FilmArray^®^ Respiratory 2.1 plus panel (Biomerieux) was used according to the manufacturer’s recommendations [58].

Briefly, approximately 300 µL of the raw sample was applied to the FilmArray pouch in an aseptic environment using sterile filter pipet tips. The pouch contains all materials and chemicals required for isolation, purification, amplification, and detection of the targeted nucleic acids. After mechanical lysis and purification using bead beating and magnetic bead technology, respectively, the total nucleic acid extract was applied to a first step multiplex PCR including reverse transcription of targets originating from RNA genomes. Subsequently, a second stage single plex PCR within a multi-well array was performed. To identify targets from positive PCR results, high resolution melting analysis was carried out harnessing the fluorescence emitted by the LCGreen Plus dye, which was captured by a charge-coupled device camera. Finally, the results of the measurement run were processed and presented by the BioFire, FilmArray 2.0, software version 2.1.336.0.

### 2.3. Statistical Analyses

Statistical analyses were performed with R [59] with the packages A Grammar of Data Manipulation (dplyr) [60] and Visualizing Categorical Data (VCD) [61]. Plots were drawn using the ggplot2 package [62]. Categorical data (infection or atopy status) were compared using Fisher’s exact test for 2 × 2 contingency tables or chi-square independence test for larger contingency tables. In three-dimensional contingency tables, Woolf’s test was used to assess if the stratifying variable influenced the odds ratio of the other two parameters. Quantitative data was analyzed using one or two-ways ANOVA tests, or Mann–Whitney U tests in the case of pairwise comparisons. In each analysis involving at least 10 comparisons or correlations, *p*-values were corrected using the FDR method. The significance threshold was set at *p* < 0.05.

## 3. Results

### 3.1. Patient Characteristics

Multiplex virus panel testing was performed in a total of 141 study subjects (*n* = 37 healthy controls, *n* = 50 wheezers, and *n* = 54 asthmatics). The mean age of the asthmatics was 9.8 years, 2.1 years in the wheezer group, and 8.2 years in the healthy control groups. The proportion of females in the diseased groups (asthmatics/wheezers) was similar (33.3% vs. 32.0%). In addition, 76.5% of asthmatics were atopic; this number was 23.4% in the wheezer group. Moreover, 42.9% of the asthmatics had reported a positive steroid intake in the past 12 months, whereas wheezers were steroid positive in 26.5% of the cases. A detailed presentation of additional clinical parameters is summarized in Table 2.

### 3.2. Rhinovirus and Respiratory Syncytial Virus Are Frequently Associated with Pediatric Exacerbation

**Figure 1:**
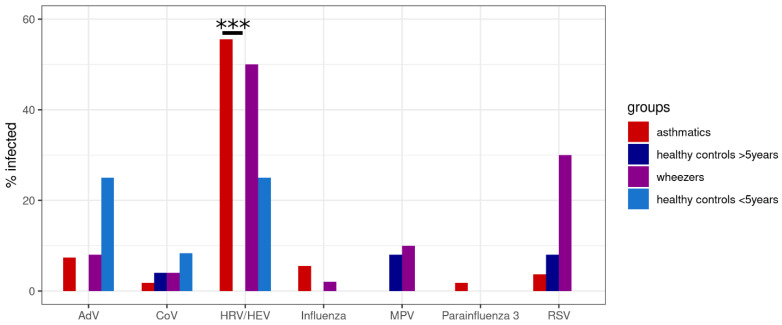
Frequency of infection in different groups. The frequency of infection with each of the studied viruses was compared between asthmatics or wheezers and age-matched controls. Rhinovirus infection levels are significantly higher in asthmatics than in older healthy controls. There is also a substantial difference in RSV infection levels between wheezers and younger controls. The pairwise comparisons were conducted with Fisher’s exact test, then the *p*-values were corrected using the FDR method. *** *p* < 0.001. Abbreviations: AdV = adenovirus, CoV = coronavirus, HRV/HEV = human rhinovirus/human enterovirus, MPV = metapneumovirus, RSV = respiratory syncytial virus).

The most frequent viruses within the study population were HRV/HEV (41.1% of all subjects were infected) and RSV (13.5%). HRV/HEV was particularly prevalent in the asthmatics group (55.6%, significant difference with age-matched controls) and RSV in the wheezers group (30%). Figure 1 shows the distribution of these viruses per group (asthmatics, wheezers, healthy controls <5 years, and healthy controls >5 years). In total, 13 subjects (9.2%) suffered from multiple infections by two viruses, 79 (56%) were infected by a single virus, and 49 (34.8%) were virus-free.

### 3.3. The Atopy Level Influences the Susceptibility of HRV Infection

**Figure 2:**
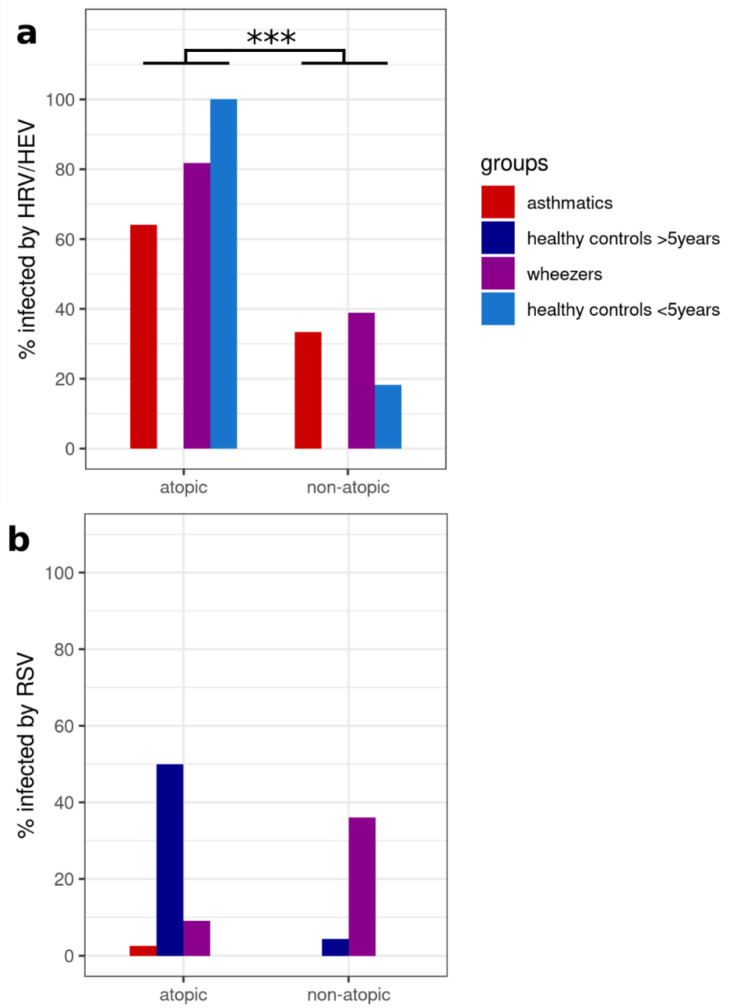
Subjects infected by HRV or by RSV as a function of atopy status. (**a**) The correlation between infection and atopic status was tested within each group or with all groups combined using Fisher’s exact test. Additionally, Woolf tests were conducted to assess if the odds ratio of infection versus atopy status differed between asthmatics and healthy controls older than 5 or between wheezers and healthy controls younger than 5. After all the tests, *p*-values were corrected using the FDR method. Atopy significantly increased HRV infection levels in all groups combined (*p* = 0.000031). (**b**) There was no significant correlation between atopy status and RSV infection levels. *** *p* < 0.001.

The patient’s atopic phenotype may influence the susceptibility to viral infection. We tested this hypothesis in our cohort by detection of HRV/HEV and RSV, the two most prevalent viruses in our cohort. For this, all HRV-infected atopic asthmatics and wheezers were grouped together and compared with those who did not have atopy but were also HRV positive. Interestingly, when all subject groups were combined, atopy was significantly and positively correlated with infectibility (*p* = 0.000031) (Figure 2).

### 3.4. Rhinovirus Infection Influences Blood Lymphocyte Levels

**Figure 3:**
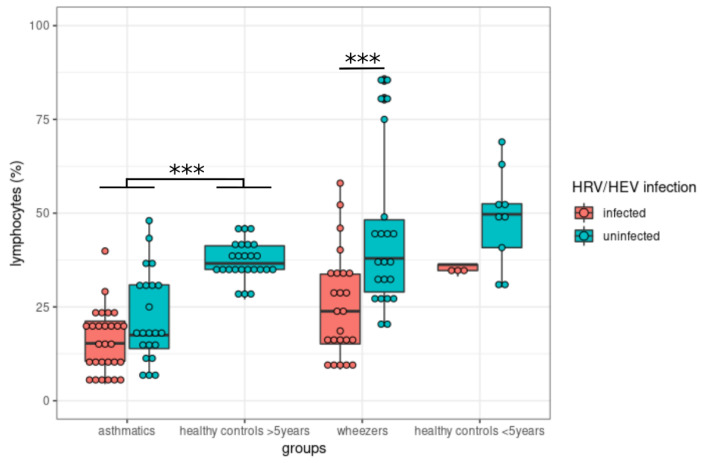
Lymphocytes decreased in HRV/HEV-infected subjects. Post-hoc pairwise comparisons of infected versus non-infected subjects were conducted within each group using the Mann–Whitney U, non-parametric test. *** = *p* < 0.001.

Currently, one of the widest and best characterized endotypes is allergic eosinophilic asthma with type 2 immune response inflammation, which stands for the type 2 T-helper cell lymphocyte (Th2) [30,63]. Chronic lower airway inflammation in asthma is caused by infiltration of inflammatory cells, including eosinophils, neutrophils, and T-helper cells, as well as mast cell activation, IgE production triggered by B lymphocytes, and epithelial cell damage [32,63,64]. We then analyzed if the group and/or viral infections influenced lymphocytes and eosinophils, which are known to be involved in atopic diseases. Results were either expressed in percentage of total leukocytes or titer per nanoliter. We restricted our analyses to HRV/HEV and RSV, the only two viruses that infected enough subjects to conduct relevant statistical analyses. Finally, we separated our dataset between asthmatics and healthy controls older than five and between wheezers and age-matched controls. Therefore, we conducted a series of 16 2-ways ANOVA tests to assess if the patients’ group, the viral infection status, or the interaction of both correlated with the selected cell subsets. *p*-values were corrected using the FDR (false discovery rate) multiple comparison correction. The percentage of lymphocytes among leucocytes was significantly correlated with HRV/HEV infection in the younger cohort (*p* = 0.0014) and asthmatic status in the older cohort (*p* = 3.4 × 10^−11^) (Figure 3).

Interestingly, the fact that a significant negative correlation was observed when all young children are combined but not within wheezers or within young controls probably indicates that the difference was related to the higher number of infected subjects within the wheezer group. This may be due to both the effects of the groups (wheezers have less lymphocytes) and of HRV infection (infected subjects have less lymphocytes), but more data need to be accumulated to be certain at this level. Furthermore, lymphocyte numbers may vary with age.

### 3.5. House Dust Mite Allergy Increases the Susceptibility to Rhinovirus Infection

**Figure 4:**
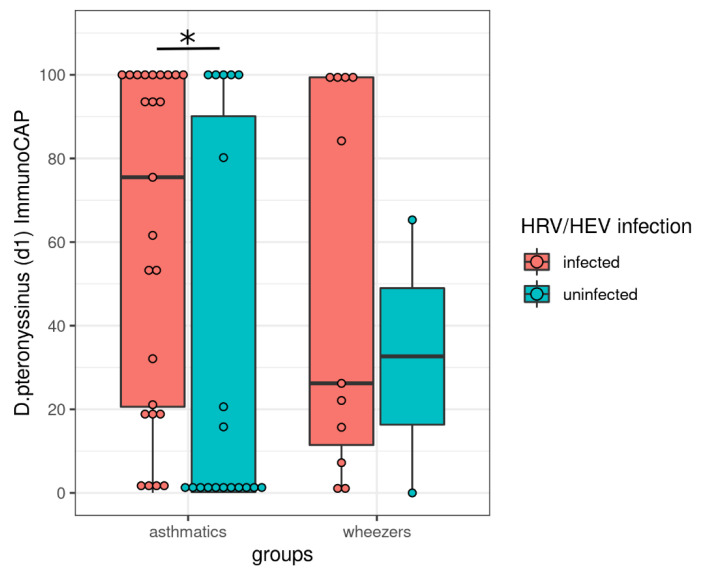
Patients with house dust mite allergies are susceptible for rhinovirus infection. We hypothesized that dust mite allergy levels, as measured by ImmunoCAP, influence the susceptibility to viral infections. We restricted our analysis to HRV/HEV and RSV and to wheezers and asthmatics since too few healthy controls accepted to undertake ImmunoCAP. For each group and each virus, we conducted a Mann–Whitney U test to assess if the viral infection status correlated with dust mite allergy levels. We found that dust mite allergies increased in HRV/HEV infected asthmatics compared with non-infected asthmatics (*p* = 0.031). * *p* < 0.05.

Our data and other studies suggest that patients with allergies or atopic diseases have an increased susceptibility to virus infections [65,66,67,68,69]. Therefore, we hypothesized that patients with house dust mite allergies had an increased rate of HRV positive test results. Here, we observed that patients with high serologic levels were at a significantly higher risk of being infected with HRV/HEV than those who showed decreased ImmunoCAP values (Figure 4).

### 3.6. The Bacterial Colonization in the Nose or Pharynx Was Not Influenced by Asthma Phenotype among Virus-Infected Subjects

To determine to what extent the microbiome profile interacts with HRV or RSV, we compared our previously published microbiome dataset [57], reanalyzed with the current virus results. We tested for the presence of different bacteria in the pharyngeal and nasal microbiome profiles of our cohort. As also described before, the most frequent bacteria were *Haemophilus parainfluenzae*, *Haemophilus influenzae, Staphylococcus aureus*, *Streptococcus pneumoniae*, and *Moraxella catarrhalis* [57]. Out of 137 tested subjects, 115 (84.4%) were colonized by at least one bacterium, and 80 (58.4%) had virus–bacteria coinfections.

**Figure 5:**
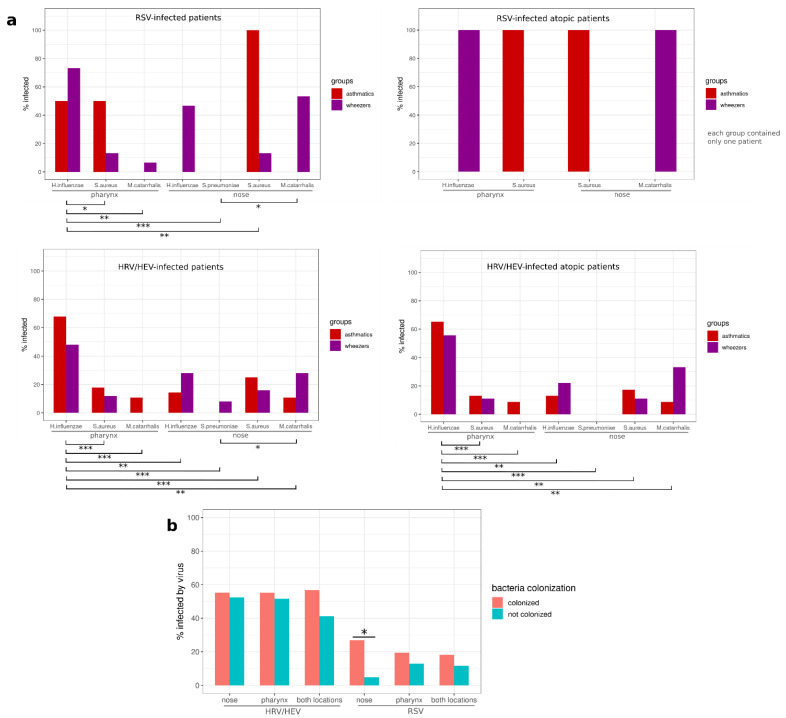
Bacterial profiles in patients with human rhinovirus (HRV) or respiratory syncytial virus (RSV) infections (**a**,**b**). In each cohort subset, we compared bacteria colonization between groups for each selected bacteria and location and between bacteria and location with all groups combined. We used chi-square tests of independence and corrected all *p*-values using the FDR method. Pairwise comparisons were conducted using Fisher’s exact test (*Haemophilus influenzae* = *Haemophilus influenzae* + *Haemophilus parainfluenzae, S. aureus = Staphylococcus aureus, S. pneumonia = Streptococcus pneumonia, M. catarrhalis = Moraxella catarrhalis).* * *p* < 0.05; ** *p* < 0.01; *** *p* < 0.001.

We hypothesized that the prevalence of these bacteria may vary between groups in RSV-infected or HRV/HEV-infected patients. However, no significant correlation between group and bacterial infection status was found in any subset of the cohort. We then compared the colonization levels of the selected bacteria in the nose or in the pharynx using the same cohort subsets and detected significant differences in RSV-infected patients (*p* = 1.45 × 10^−4^), HRV-infected patients (*p* = 3.68 × 10^−9^), and HRV-infected atopic patients (*p* = 1.30 × 10^−8^) (Figure 5a). Interestingly, pharyngeal *Haemophilus (para-)influenza* were detected in more patients than all other bacteria and were also more prevalent than nasal *Haemophilus (para-)influenza*.

We also asked the question of whether colonization by pathogenic bacteria was associated with HRV/HEV or virus infection. For each of these two viruses, we compared virus infection counts in patients colonized by *Haemophilus influenzae/parainfluenzae*, *Staphylococcus aureus*, *Streptococcus pneumoniae*, or *Moraxella catarrhalis* in the nose, in the pharynx, or in any of these two locations. We found that RSV infection was significantly increased in subjects with nose colonization (*p* = 0.016, fisher’s exact test) (Figure 5).

## 4. Discussion

Asthma is categorized into different phenotypes and endotypes [70]. Substantial efforts are made to prevent subjects from developing atopic disorders and even predict a severe course or an exacerbation [71,72,73,74,75,76,77,78,79]. The novel therapeutic alternatives have significantly improved the clinical course of asthma symptoms [80,81,82,83]. Beside the allergen aspects as triggering factors for exacerbation, viruses play a major role in seasonal exacerbations, e.g., in winter [17,55,77,84,85,86,87]. An infection with HRV or RSV may be associated with life-threatening events and affect disease development and progression [88,89,90,91,92,93,94,95]. Interestingly, in our cohort, HRV and RSV were the most prevalent viruses, which were detected in the nasopharyngeal swab specimens obtained during an exacerbation. It can be proposed that these viruses are the causative pathogens of the current exacerbation.

Due to improved techniques, e.g., multiplex-PCR, it is possible to diagnose examination results rapidly, which enables earlier initiation of therapies [58,96,97]. Such techniques also have the advantage of detecting subgroups of viruses, which can lead to important clues about the corresponding pathomechanism [58]. Indeed, more clinical and experimental information are needed to establish vaccination strategies to counteract the prevalence of atopic diseases. In this project, we were able to detect multiple respiratory viruses and bacteria in the nasopharyngeal swabs of our study population during exacerbation in panel studies, and this data can be utilized for further studies and biomarker development. Such observations are important as they lead to insights into the exacerbation of the diseased child and lay fundamental milestones for further translational projects. In detail, we demonstrated that children with asthma have increased susceptibility to HRV. Our data confirm previous work, and similar studies also present a correlation between HRV and exacerbation [90,98].

HRV, as a single-stranded RNA virus, is an enterovirus and belongs to the picornavirus family, where its capsid has four viral proteins [45,99,100,101,102]. It is classified into three species, RV-A, -B, and -C, and it enters the airway epithelial cells through receptor-mediated endocytosis by activating I-CAM 1 (RV-A/-B) and LDLR (RV-A/-B), whereas RV-C binds to the CDHR3 receptor [99,103,104]. Upon entering, the genome is recognized by pattern recognition receptors, including Toll-like receptors. Subsequently, distinct immune responses are induced by secreting chemokines and cytokines [99,100,101,105,106]. Epithelial cells infected with HRV show a predisposition towards bacterial colonization, e.g., *Staphylococcus aureus* and *Streptococcus pneumoniae* [99]. Asthmatics infected with HRV have a more severe course than healthy patients, and in concordance with our work, previous studies have already presented that an early infection is a risk factor for children with atopic predispositions (e.g., allergic sensitization, genetic predisposition, etc.) [38,88].

In addition, an early infection with RSV or HRV can cause bronchiolitis and lead to serious damage of the epithelium, which is also associated with an increased risk of developing asthma in later years [17,37,107]. For example, Bergroth and colleagues have shown that children with previous RV-A or RV-C bronchiolitis need to take asthma medications at an early phase and several years after infection [37]. Longitudinal and follow-up analyses of our cohort over several years should indicate whether a previous exacerbation with RSV or HRV may have a negative impact on the course of the subjects.

The respiratory tract harbors not only viruses, but also other microorganisms, such as bacteria and fungi [108,109,110,111,112,113]. The totality of all microorganisms is called the microbiome, and its pathogenic relevance is still investigated in different molecular and clinical contexts [113,114,115,116,117,118,119]. Several studies have already delineated the relationship between the airway microbiome and the pathogenesis of asthma [113,119,120,121,122,123]. Importantly, the first bacterial colonization in humans occurs within 24 h post-partum and is delayed when the child is delivered by cesarean section [113,124]. Due to various external influences, the microbiome changes throughout the life [125]. Viral infections that have passed through may also have an impact on the composition of the microbiome [126]. In our previous work, we observed that the wheezer cohort showed more colonization with distinct bacteria, including *Moraxella catarrhalis* and *Haemophilus (para-)influenzae*, whereas asthmatics had an increased abundance of *Staphylococcus* and *Streptococcus* in the nasopharyngeal swabs [57]. Interestingly, Teo and colleagues showed that patients with a colonization of *Streptococcus*, *Moraxella,* and *Haemophilus* have an increased risk of infection with respiratory viruses, particularly when such patients with the combination of RSV and *Moraxella* suffered from severe symptoms [127]. Further works also highlighted the high risk of being hospitalized for those patients with a *Haemophilus*, *Moraxella*, and *Streptococcus* colonization and an RSV infection [128].

In addition to RSV, it was demonstrated that HRV also plays a special role in bronchiolitis [88,129]. Furthermore, it was shown that patients with *Moraxella* colonization have a higher risk of being infected with HRV [130]. For example, Rosas-Salazar and coworkers found that children with HRV and RSV infections were predominantly colonized with *Moraxella*, *Streptococcus*, *Corynebacterium*, *Haemophilus*, and *Dolosigranulum* [130].

Considering these studies, our results also support the hypothesis that a colonization with bacteria may be associated with an increased risk of respiratory virus infections. Here, we observed that patients with positive test results of HRV/HEV and RSV infections also had colonizations of distinct bacteria in nasopharyngeal swabs specimens. Therefore, compared to previously published data, the increased detection of HRV and RSV in our cohort provides a good correlation to previous studies. Nevertheless, it is difficult to classify the extent to which the microbiome is a platform for viral infections or whether the microbiome level affects the infectivity, the disease duration, and the severity of respiratory infection in a patient.

Interestingly, patients with high ImmunoCAP values *(Dermatophagoides pteryonossinus)* were at higher risk of being HRV positive than those with low values. Interestingly, for SARS-CoV-2, there are contradictory results for asthmatics. For example, Yan and colleagues observed that patients with asthma histories suffer from severe SARS-CoV-2 courses. They observed in their Korean cohort that patients with asthma and allergic rhinitis had a serious clinical outcome [69]. Importantly, in the review of Chatziparasidis and Kantar, the authors summarized the fact of why children with asthma may not belong to the high-risk groups in terms of SARS-CoV-2 infections [22]. Indeed, the majority of the patients infected with SARS-CoV-2 were either asymptomatic or had few serious symptoms [22]. Subsequently, Radzikowska et al. analyzed the expression of receptors targeting SARS-CoV-2 in different samples derived from diseased patients (including asthmatics) and healthy controls. They observed that asthmatics had a higher expression of CD147- and ACE2-related genes in different sample types. Whether this or other factors are the reasons for different SARS-CoV2 morbidity will be interesting to investigate in further studies [26].

Finally, it remains to be ascertained as to what extent viral infections affect microbial composition or, conversely, to what extent the microbiome causes an increased susceptibility to viral infection [113]. This pathomechanism is not yet fully understood and will be part of our follow-up work.

## 5. Limitations

As also discussed previously in the study protocol [52], subjects from the western part of Germany were included here while disregarding the aspect of different living conditions, e.g., urban vs. rural. The fact that we recruited a local cohort prevents us from investigating potential external influences on child exacerbation pathomechanisms. Nevertheless, this aspect implies that the cohort is more coherent with less confounding factors, which strengthens our conclusions regarding potential mechanisms [52]. As described previously, due to an improved and updated analysis panel, a specification of HRV and enterovirus is still not possible and a potential cross-reaction with enteroviruses cannot be excluded at this level [58]. Moreover, the longitudinal observational aspect of our subjects over several years is currently missing to address whether children with a history of RSV or HRV infection have had an atopic progression in later years of age. Nonetheless, the recruitment of exacerbated patients is a major hurdle for clinicians and scientists. Thus, this local cohort provides important information on the current status of affected children, where the obtained biomaterials are valuable to study experimental questions and to correlate these results with the clinical dataset for the purpose of deciphering important information for potential prevention strategies in clinical settings. Thus, further experimental work should study the impact of HRV subtypes in nasal epithelial cell cultures obtained from patients during exacerbation and the response against the virus in these cultures.

## 6. Conclusions

Asthma is a multifactorial and heterogeneous disease with different phenotypes and endotypes. Several trigger factors are involved during asthma development and exacerbation, and distinct models have already shown the underlying signaling processes. Importantly, viruses are particularly involved, and HRV takes a major part in causing an acute asthma exacerbation. Here, HRV was common in the nasopharyngeal swab specimens of our entire cohort where patients suffered from serious exacerbation event. It would be interesting to study the role of viral infections during the SARS-CoV-2/COVID-19 pandemic and during asthma exacerbation/wheezing. The study inclusion of the last patient was in the year 2019 before the pandemic, so this hypothesis cannot be tested here. Complementary, observational studies in childhood should be performed soon to analyze both seasons. Thus, many efforts need to be invested into the development of vaccination and prevention strategies against HRV to protect asthmatics from such a deteriorated status, which has significant effects on the quality of life.

## Figures and Tables

**Table 1:** Respiratory virus and bacteria panel for studying the pathogenome of nasopharyngeal swab specimens of the study cohort.

AdV 2 AdV 3 AdV 6 AdV 7.1 AdV 8	CV 229E CV HKU1 CV NL63 CV OC43	MERS-CoV SARS-CoV2	HMPV	HRV/HEV	Influenza A Influenza B	PIV 1 PIV 2 PIV 3 PIV 4	RSV
*B. p.* *B. pp.*	*C. pneumoniae*	*M. pneumoniae*					

Abbreviations: AdV = adenovirus; CV = coronavirus; MERS-CoV = Middle East respiratory syndrome coronavirus; HMPV = human metapneumovirus; HRV/HEV= human rhinovirus/enterovirus; PIV = parainfluenza virus; RSV = respiratory syncytial virus; *B. p.* = *Bordetella pertussis*; *B. pp.* = *Bordetella parapertussis*; *C. pneumoniae* = *Chlamydia pneumoniae*; *M. pneumoniae* = *Mycoplasma pneumoniae*; SARS-CoV2 = severe acute respiratory syndrome-coronavirus type 2.

**Table 2:** Descriptive summary of patient characteristics.

	Asthmatics	Wheezers	Healthy Controls
Population (*n*)	54	50	37
Age (years) average (minimum-maximum)	9.8 (5.29–17.27)	2.1 (0.43–4.48)	8.2 (1.39–16.30)
Female (*%*)	33.3	32.0	45.9
Positive atopic status (*%*)	76.5 (*n* = 51)	23.4 (*n* = 47)	8.1
Negative steroid status (*%*)	57.1 (*n* = 49)	73.5 (*n* = 49)	0
Breast feeding (*%*)	76.7 (*n* = 43)	72.9 (*n* = 48)	91.2 (*n* = 34)
Maternal alcohol/tobacco abuse (*%*)	20.5 (*n* = 44)	22.9 (*n* = 48)	5.9 (*n* = 34)
Pet owner (*%*)	23.3 (*n* = 43)	21.3 (*n* = 47)	29.4 (*n* = 34)
Mold exposition (*%*)	50.0 (*n* = 42)	28.3 (*n* = 46)	17.7 (*n* = 34)
Traffic exposition (*%*)	39.5 (*n* = 43)	34.0 (*n* = 47)	14.7 (*n* = 34)

## Data Availability

The data presented in this study are available on request from the corresponding authors.
